# Acupuncture for uremic pruritus: A systematic review and meta-analysis protocol

**DOI:** 10.1371/journal.pone.0313403

**Published:** 2024-11-08

**Authors:** Ning Gao, Lei Wang, Weiming Wang, Yufeng Guo

**Affiliations:** 1 Department of Acupuncture, Guang’anmen Hospital, China Academy of Chinese Medical Sciences, Beijing, China; 2 Department of Dermatology, China-Japan Friendship Hospital, Beijing, China; MOH Holdings Pte Ltd Singapore, SINGAPORE

## Abstract

**Background:**

Uremic pruritus (UP) or chronic kidney disease-associated pruritus (CKD-aP) is one of the most intractable dermatologic symptom in patients with chronic kidney disease. Several randomized controlled trials (RCTs) have been conducted to investigate the antipruritic effects of acupuncture on UP/CKD-aP and suggested a significant therapeutic effect, while the evidence supporting the application of acupuncture is limited.

**Objectives:**

This study will assess the efficacy and safety of acupuncture for patients with UP/CKD-aP.

**Methods:**

*Data Sources*: RCTs will be searched in MEDLINE, EMBASE, the Cochrane Central Register of Controlled Trials, Web of Science, the Chinese Biomedical Literature Database, the China National Knowledge Infrastructure, Wanfang Database, VIP Database, the WHO International Clinical Trials Registry Platform portal and www.ClinicalTrials.gov from inception to 31st August 2024. *Study eligibility criteria*: RCTs in English and Chinese conducted on UP/CKD-aP patients will be included. *Participants*: Adult patients diagnosed with UP/CKD-aP will be included. *Interventions*: All acupuncture interventions in the management of UP/CKD-aP will be included, compared with no treatment, placebo or sham acupuncture, or other treatment agents. *Outcome measures*: The primary outcome will be the change in the severity of itching evaluated by validated scales. *Study appraisal and svnthesis methods*: If necessary, a meta-analysis will be performed for the pooled therapeutic effect by Review Manager 5.3, or a qualitative descriptive analysis will be presented. The data will be transformed into the risk ratio (RR) for binary data and the mean difference (MD) or standardized MD for continuous data for analysis.

**Results:**

This review will update evidence of RCTs evaluating acupuncture for UP/CKD-aP.

**Limitations:**

Anticipated challenges contain the methodological and clinical heterogeneity in terms of evaluation tools and acupuncture interventions within included studies.

**Conclusion and implications:**

It will benefit patients and impact health-care decision-making regarding the models of care that are feasible for patients.

**Trial registration:**

**PROSPERO CRD42021257001**.

## Introduction

Uremic pruritus (UP) or chronic kidney disease-associated pruritus (CKD-aP) is a common and burdensome symptom in patients with end-stage kidney disease (ESKD) or chronic kidney disease (CKD) [1]. It has been reported that the incidence of UP/CKD-aP range from 15% to 49% [2] before dialysis, and 50% to 90% [3] during continuous dialysis treatment, with a heavier burden in South Asia [4,5]. Although this type of itching is often referred to as UP/CKD-aP, it can also occur in patients who are not yet on dialysis [6], without other symptoms related to uremia. Therefore, it is also known as CKD-aP [7]. The clinical manifestations of UP/CKD-aP typically include bilateral symmetric pruritus, most commonly affecting the back, arms, head, and abdomen [8,9]. The patchy features include dry, scaly skin that appears similar to the skin of individuals who do not suffer from pruritus [7], and the itching can be localized or generalized, continuous or intermittent, with increasing intensity at night [10]. At the same time, the above suffering is strongly associated with sleep disturbance, negative emotions, resulting in declined quality of life and increased mortality [11]. The precise mechanisms involved in the development of UP/CKD-aP are unclear but likely multifactorial in nature. The immune hypothesis [12,13] and opioid hypothesis [14,15] have been proposed as the potential pathogenesis of UP/CKD-aP. Many other triggering factors like the abnormal serum electrolytes metabolism [16–18], inadequate dialysis [19], hyperparathyroidism [20], xerosis caused by sweat gland atrophy [21] were also hypothesized.

As of now, treatment of UP/CKD-aP is still a frustrating endeavor, and no clear consensus exists regarding the optimal management strategy due to inadequate knowledge and understanding in terms of underlying pathophysiological mechanisms [22]. Optimizing the dialysis modality, skin rehydration therapy, and nutrition have been recommended as the initial treatment of a stepwise management for UP/CKD-aP. Supportive therapies containing optimization of calcium and phosphorus levels, oral antihistamines, or pregabalin, are marginally effective [23]. Some promising agents(e.g. gabapentin) still need cost-effectiveness assessments of their safety and availability [24]. Thus, it is necessary to explore novel therapies for UP/CKD-aP.

Acupuncture refers to the practice of inserting needles into anatomical locations on the body surface for therapeutic purposes, based on the concept of “Meridians” originated in traditional Chinese medicine [25]. It has been widely used for dermatologic conditions complaining of pruritus around the globe [26], irrespective of the causes [27,28]. The mechanism of acupuncture for pruritus may involve the influence on the endogenous opioid system to inhibit the afferent fibers of pruritogenic impulses and inhibiting the proliferation of histamine and mast cells [29]. Two RCTs investigating the antipruritic effects of acupuncture on histamine-induced itch in healthy volunteers suggested a significant therapeutic effect of acupuncture [30,31]. Moreover, several RCTs and four systematic reviews have been published to investigate the efficacy and safety of acupuncture for treating UP/CKD-aP [32–35]. Among these, two reviews—one from 2010 [34] and another from 2021 [32]—offered descriptive qualitative summaries. In contrast, a separate review, which excluded Chinese databases, synthesized findings from three non-randomized controlled trials conducted prior to 2017, ultimately yielding inconclusive results. In 2023, Zhang et al [33]. conducted a meta-analysis, wherein the primary outcome measure selected was the efficacy rate, defined as the percentage of patients who were clinically cured, reported the treatment as markedly effective, or reported the treatment as effective. The limitation of this selection lies in the fact that the efficacy rate is neither a validated assessment metric nor a universally endorsed method for quantifying CKD-aP [36]. As a result, the conclusions derived may not adequately substantiate the effectiveness of acupuncture in the treatment of UP/CKD-aP. The incorporation of more recent randomized controlled trials (RCTs) enables our study to address existing gaps in the literature by providing a novel perspective and enhancing the understanding of the potential effectiveness of acupuncture in the management of UP/CKD-aP [37]. This review will build on the findings of the previous reviews and expand the breath and scope of the knowledge of acupuncture for UP/CKD-aP in ESKD or CKD patients.

## Methods

### Criteria for considering studies for this review

#### Types of studies

Randomized controlled trials in English and Chinese conducted on patients diagnosed with UP/CKD-aP will be included. Specifically, studies will be included if they assess the outcomes of acupuncture treatment for uremic pruritus at baseline and at least one follow-up timepoint. There will be no restrictions on publication status. We will remove uncontrolled clinical trials, quasi-RCTs, non-RCTs, and animal studies.

#### Types of participants

Adults patients (≥18 years old) diagnosed with UP/CKD-aP will be included [36,38], without limitations on sex, race, CKD stages, or whether they have received dialysis.

#### Types of interventions

We will include all acupuncture interventions in the management of UP/CKD-aP, compared with no treatment, placebo or sham acupuncture, or other treatment agents. Acupuncture treatments are defined as different types of acupuncture operations for simulating acupoints, which contain manual acupuncture, electroacupuncture, acupressure, auricular needles, auricular-plaster, and so on. This review will assess the following comparisons: acupuncture versus no treatment; acupuncture versus placebo or sham acupuncture; acupuncture plus one or more therapies with sham acupuncture plus the same therapies; acupuncture versus routine treatment, or other agents; acupuncture plus another therapy versus the same treatment alone. RCTs comparing different acupuncture treatments will be removed.

### Types of outcome measures

#### Primary outcomes

Changes in the severity of itching evaluated by participants using validated scales [36,39], e.g., Visual Analogue Scales, Numerical Rating Scales, the Verbal Rating Scale and a question from the Kidney Disease QoL-Short Form or urdu 5D-Itch scale.

#### Secondary outcomes

1. Change in the whole condition of pruritus (e.g., the area, distribution, frequencies).
2. Patient global evaluation of improvement (e.g., the effective rate).
3. Change in quality of life evaluated by any validated implements.
4. Measurement of recurrent rate of uremic pruritus (recurrence of pruritus symptoms during the follow-up period).
5. Reduction in the use of medications or modalities required to alleviate itch.
6. Adverse events relating to acupuncture as reported in the included studies.

### Search methods for identification of studies

#### Electronic searches

We will search MEDLINE, EMBASE, the Cochrane Central Register of Controlled Trials, Web of Science databases, the Chinese Biomedical Literature Database, the China National Knowledge Infrastructure, Wanfang Database, VIP Database, as well as the WHO International Clinical Trials Registry Platform portal and www.ClinicalTrials.gov from inception to 31st August 2024. The following terms will be used for searching: Uremia [MESH], entry terms: uremi*, uraemi*; pruritus [MESH], entry terms: pruritis, UP, uremic pruritus, itch, itching; renal insufficiency, chronic [MESH], entry terms: chronic renal insufficiencies, chronic renal disease, chronic kidney disease, chronic kidney disease-associated pruritus, CRD, CKD, CKD-aP; kidney failure, chronic [MESH], entry terms: chronic renal failure, chronic kidney failure, CRF, CKF, end-stage kidney disease, end-stage renal disease, ESRD, ESKD, ESRF, ESKF; renal dialysis [MESH], entry terms: hemodialysis, haemodialysis, hemofiltration, haemofiltration, dialysis, peritoneal dialysis; acupuncture [MESH], acupuncture, ear [MESH], acupuncture points [MESH], acupuncture therapy [MESH], entry terms: electroacupuncture, acupoint*, acupuncture therapy, auricular-plaster, transcutaneous electric nerve stimulation, ear acupuncture, acupressure, auricular needle, fire needling, warm needling, pyonex, dermal needle, electric stimulation therapy; randomized controlled trial [MESH], entry terms: controlled clinical trial, randomized, placebo, randomly, trial and groups (The search strategy for the Web of Science database is shown in Table 1).

**Table 1:** Searching strategy for Web of Science.

**Database**	**Search strategy**
Web of science Search: 8,285	TS = ("randomized controlled trial" OR "controlled clinical trial" OR randomized OR placebo OR randomly OR trial OR groups) AND (TS = ("electroacupuncture" OR "acupoint*" OR "acupuncture therapy" OR "auricular-plaster" OR "transcutaneous electric nerve stimulation" OR "acupuncture" OR "acupressure" OR "auricular needle" OR "fire needling" OR "warm needling" OR "ear acupuncture" OR "pyonex" OR "dermal needle” OR "electric stimulation therapy") ) AND (TS = ("UP" OR "uremic pruritus" OR "chronic kidney disease-associated pruritus" ((" chronic renal disease" OR "chronic kidney disease" OR "CRD" OR "CKD" OR "chronic kidney insufficiencies" OR "chronic renal insufficiencies" OR "chronic renal failure" OR "chronic kidney failure" OR "CRF" OR "CKF" OR "end-stage renal" OR "end-stage kidney" OR "ESRD" OR "ESKD" OR "ESRF" OR "ESKF" OR "uremi*" OR "uraemi*" OR "hemodialysis" OR "haemodialysis" OR "hemofiltration" OR "haemofiltration" OR "dialysis" OR "peritoneal dialysis") AND (pruritus OR itch OR itching))))索引 = SCI-EXPANDED, SSCI, A&HCI, CPCI-S, CPCI-SSH, BKCI-S, BKCI-SSH, ESCI, CCR-EXPANDED, IC 時間範圍 = 所有年份(1985–2024

### Searching other resources

We will also search the bibliographies of included studies, previously published reviews, and Google Scholar for potentially eligible articles.

### Data collection and analysis

#### Selection of studies

**Fig 1:**
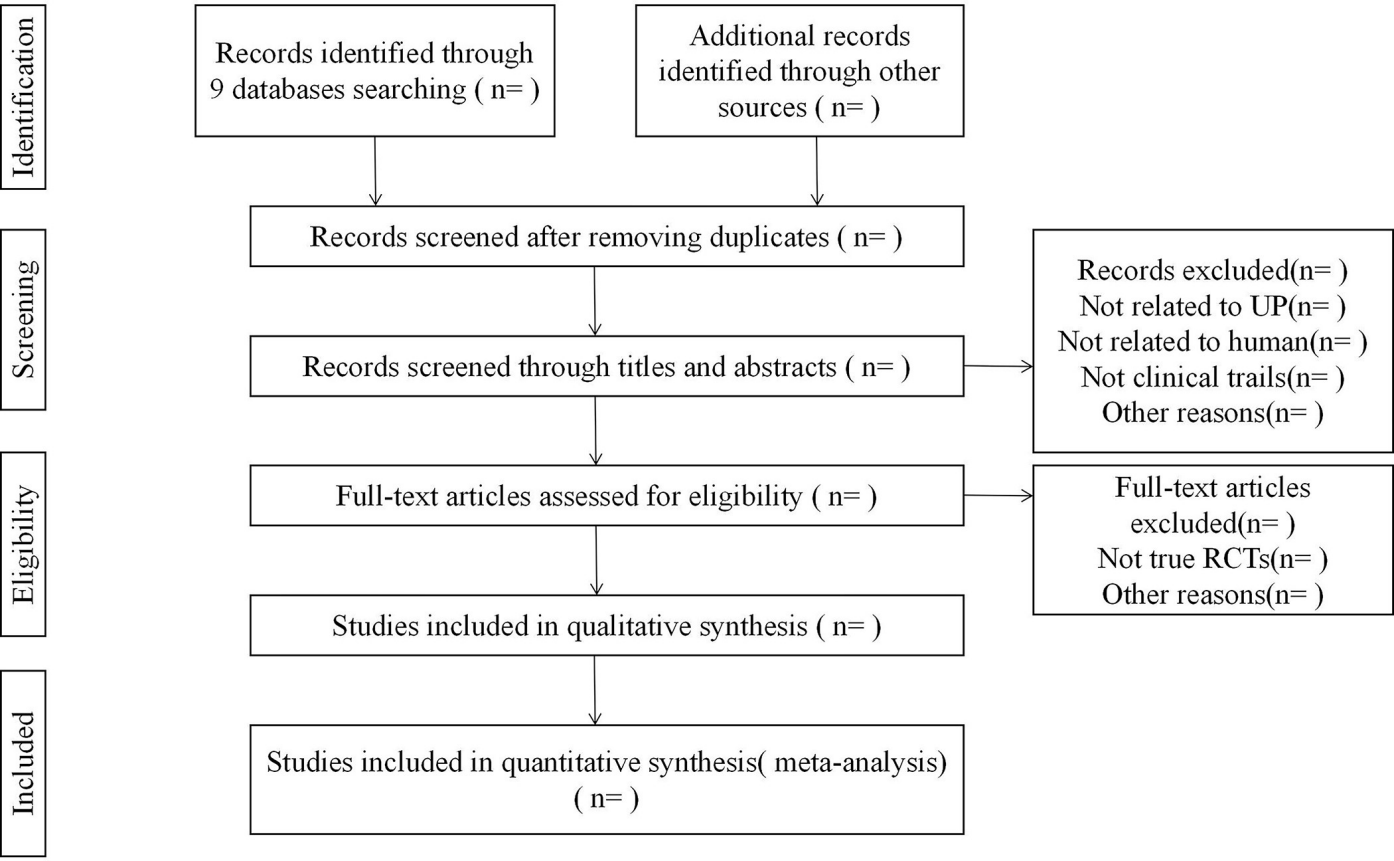
Flow diagram of study selection process.

Two independent reviewers (Ning Gao and Lei Wang) will examine titles and abstracts of all studies achieved and remove irrelevant literature. The full text of possibly relevant trials will be obtained for further evaluation based on the predefined criteria. Discrepancies will be arbitrated by Weiming Wang and resolved by consensus. The details of trial selection and excluded reasons will be recorded in a PRISMA flow chart (Fig 1).

### Data extraction and management

A predetermined data extracted table will be designed, containing the following sections: article identification (author(s)/ year of publication, country); methodological characteristics (design, sample size, loss to follow-up; inclusion and exclusion criteria); participants data (number of patients by gender, age, duration of symptoms, kidney disease diagnosis, and stage); description of interventions (treatment frequency, duration, number and location of acupoints); outcomes measure (types of outcomes, measuring time points, measuring tools, follow up information); data analysis; reported results; adverse events. Before beginning formal extraction, two reviewers will do calibration exercises to ensure consistency across reviewers. A third researcher will cross-check the extracted data to reach a final consensus.

### Assessment of risk of bias in included studies

Two reviewers (Ning Gao and Lei Wang) will separately assess the risk of bias for included trials based on the Cochrane Collaboration’s tool. The following aspects will be evaluated: selection bias, performance bias, detection bias, attrition bias, reporting bias, and other potential deviations. The risk of bias for each section will be classified into the following levels: ‘low risk,’ ‘high risk,’ or ‘unclear.’ The quality of evidence will be evaluated via the Grading of Recommendations Assessment, Development and Evaluation instrument, divided into 4 levels: high, moderate, low, and very low. Where a consensus cannot be achieved, a third researcher will make the final decision. The information of included trials on the risk of bias as well as evidence quality assessment will be presented in tabular form.

### Measures of treatment effect

Data in terms of effectiveness will be synthesized and analyzed by RevMan V.5.3. For continuous variables, the standardized mean difference (SMD) or mean difference (MD) will be used with 95% confidence intervals (CIs) to measure the treatment effect. Dichotomous data will be analyzed through a risk ratio (RR) with 95% CIs. When significant heterogeneity is detected, a random-effects model will be used, a fix-effects model otherwise.

### Unit of analysis issues

Data from parallel design studies will be pooled for meta-analysis. The unit of analysis will be aggregated outcome data in the included studies because of the lack of individual patient data.

### Dealing with missing data

On condition that some data are missing or insufficient, we will attempt to contact the original author to request numerical outcome data or analyze existing data based on an intent-to-treat principle. A sensitivity analysis will also be conducted to examine the influence of missing data on the effect size.

### Assessment of heterogeneity

Both the χ^2^ test and *I*^2^ statistics will be performed in the forest plot using RevMan V.5.3 for the assessment of heterogeneity. A fixed-effect model will be used to pool data if there is no obvious heterogeneity (*I*^2^<50% and *P* >0.1), or we will adopt a random-effects model. If there is substantial unexplained heterogeneity, a prespecified subgroup analysis will be conducted to investigate sources of heterogeneity.

### Assessment of reporting bias

If 10 or more studies are synthesized in the meta-analysis, a funnel plot will be constructed to examine possible reporting bias.

### Data synthesis

Treatment effects across studies will be calculated in RevMan 5.3 software. The fixed-effects or random-effects model will be chosen depending on heterogeneity. The MD or SMD with 95% CIs will be used to express continuous variables, and RR with 95% CIs for dichotomous variables. If the characteristics of included trials are not similar enough for pooling to make sense, we will present a narrative review of individual trials. It will be deemed statistically significant if *P*<0.01.

### Subgroup analysis and investigation of heterogeneity

If the necessary data are available, we will perform subgroup analysis based on the heterogeneity of primary diseases; severity of symptoms; the acupuncture treatments’ types (including manual acupuncture, electroacupuncture, acupressure, auricular needle, or auricular-plaster); different types of control groups (sham acupuncture, traditional agents, no treatments, with or without concomitant treatments) and other clinical differences.

### Sensitivity analysis

We will perform sensitivity analysis for the primary outcome in terms of the following aspects: studies with small sample sizes, differences in methodological quality and statistical model (random-effects or fixed-effects model).

### Patient and public involvement

Not applicable. This protocol of systematic review and meta-analysis does not directly target individuals of the public. Data will be pooled from published trials achieved from the above-mentioned databases as well as manual searching.

### Ethics and dissemination

Formal ethical approval is not needed, since extracting data will not be related to privacy. The results of the systematic review will be disseminated via peer-reviewed publication or a relevant conference report.

## Discussion

UP/CKD-aP has been identified as an independent risk factor for depressive symptoms and mortality in patients with CKD or ESKD [2,40]. Clinicians tend to underestimate the prevalence and the impact of UP on their patients, as UP/CKD-aP is not a quite lethal condition when compared with other comorbid conditions accompanied by CKD or ESKD [41]. At present, the definite pathogenesis of UP/CKD-aP is unknown, thus effective treatment of UP/CKD-aP remains a significant therapeutic challenge [42]. A stepwise approach(e.g., systemic H1 antihistaminics, topical corticosteroids, gabapentin or pregabalin, antidepressants) is suggested in choosing a therapeutic modality for the itching symptom [43,44]. However, some drugs(e.g. gabapentin, pregabalin) in stepwise approach are totally dependent on renal elimination, their significantly increased half-life in hemodialysis patients is noteworthy [43,45]. Thus, it is still need assessments of these agents to determine whether the widespread use of them is feasible, especially in patients with renal failure and continuous hemodialysis, which have potential risks of aggravating the renal injury [43]. Acupuncture is a kind of external therapy with high security and involves a broad indication spectrums [46]. It is indicated that acupuncture activates small diameter afferent nerve fibres and produces therapeutic effects on the associated visceral organs, which is likely due to the release of endogenous opioids [47]. Furthermore, acupuncture may decreases urinary albumin secretion, improves renal blood flow and glomerular filtration rate in nephropathy patients or animals [48]. Meanwhile, it has also been suggested for many other comorbidities management in patients with CKD like renal function improvement [29,42]. Acupuncture might be a promising technique to manage UP/CKD-aP in patients with CKD or ESKD.

Although previous reviews [32–35] have already synthesized partial RCTs separately, the evidence of acupuncture for UP/CKD-aP is still inconclusive. This systematic review is of considerable significance for several reasons. Firstly, by incorporating recently published randomized controlled trial [37], our study aims to provide an updated assessment of the efficacy of acupuncture in treating uremic UP/CKD-aP. This could potentially yield more reliable evidence regarding the recommendation of acupuncture as a therapeutic option for patients suffering from UP/CKD-aP. Secondly, in contrast to recent meta-analyses that predominantly concentrate on effectiveness rates as the principal outcome, this study prioritizes alterations in pruritus severity as the primary endpoint. Pruritus severity, a widely endorsed quantitative measure for UP/CKD-aP, offers a more direct and efficacious means of quantifying these conditions. Furthermore, this study integrates other widely utilized assessments, including the Dermatology Life Quality Index (DLQI), as secondary outcomes. This approach ensures that the conclusions more comprehensively and accurately represent the effects of acupuncture on the condition.

Anticipated limitations and challenges include the following issues. To begin with, the assessment tools and acupuncture interventions in the included studies may exhibit methodological and clinical heterogeneity. Secondly, including only studies published in Chinese and English may increase bias. At last, several scales for measuring UP/CKD-aP originating from the English version have not been validated after translation into Chinese. We will thus explain the results with caution and adopt a rigorous approach when evaluating the overall evidence.
